# Urinary marker of oxidative stress in children correlates with molecules in exhaled breath

**DOI:** 10.3389/fmolb.2025.1511119

**Published:** 2025-03-26

**Authors:** Amanda Gisler, Kapil Dev Singh, Andrea Marten, Fabienne Decrue, Urs Frey, Pablo Sinues, Jakob Usemann

**Affiliations:** ^1^ University Children’s Hospital Basel UKBB, University of Basel, Basel, Switzerland; ^2^ Department of Biomedical Engineering, University of Basel, Basel, Switzerland

**Keywords:** urine, oxidative stress, exhaled breath, child, smoking

## Abstract

Real-time breath analysis has shown potential as a non-invasive method for detecting oxidative stress and airway inflammation. However, there is a lack of data on the association of full-breath profiles with established urinary biomarkers of oxidative stress and respiratory inflammation, which could help advance the implementation of this method in clinical practice. We analyzed breath profiles of 25 tobacco smoke-exposed and 103 non-exposed children via real-time secondary electrospray ionization high-resolution mass spectrometry (SESI-HRMS) and determined in parallel the urinary concentrations of biomarkers of oxidative stress and respiratory inflammation. We evaluated the correlation between breath features and urinary biomarkers and tested the prediction of these biomarkers by exhaled breath. We found 71 breath features that correlated significantly with the urinary oxidative stress marker 8-iso-prostaglandin F2α (8-iso-PGF2α). The agreement (mean ± standard deviation) (Lin’s concordance correlation) between breath-predicted and actual urinary 8-iso-PGF2α levels was 0.37 (0.05). In conclusion, our results suggest that the real-time breath analysis via SESI-HRMS has promising potential to gauge oxidative stress.

## Introduction

Oxidative stress and airway inflammation increase the risk for the development and progression of respiratory disease in children (Cheraghi and Salvi, 2009; Noutsios Georgios and Floros, 2014). Therefore, sensitive diagnostic methods are needed to detect these biochemical processes. However, most established methods for detecting oxidative stress or airway inflammation are invasive or have limited specificity for the airways, requiring induced sputum, tissue, blood, or urine samples. Breath-based analytical methods offer an attractive opportunity for non-invasive and lung-specific assessments of oxidative stress (Amann et al., 2014) and airway inflammation (Dweik et al., 2011) at the molecular level. Nevertheless, except for the measurement of exhaled nitric oxide (FeNO) to assess endobronchial inflammation, the majority of these methods are not yet used in clinical practice. Particularly attractive are breath analysis methods that allow real-time and parallel evaluation of multiple biomarkers without any sample pretreatment that could compromise the results (e.g., proton-transfer-reaction mass spectrometry, PTR-MS (Blake et al., 2009); selected-ion flow-tube mass spectrometry, SIFT-MS (Španěl and Smith, 2011); and secondary electrospray ionization high-resolution mass spectrometry, SESI-HRMS (Li et al., 2006)). Previous studies have already shown that features related to oxidative stress and airway inflammation can be detected in breath using such real-time breath analysis methods (García-Gómez et al., 2015). However, no study to date has further explored the potential of real-time breath analysis to detect oxidative stress and airway inflammation by parallelly assessing breath features and already established urinary biomarkers for these processes (e.g., 8-iso-prostaglandin F2α (8-iso-PGF2α) (van 't Erve et al., 2015; Zhang et al., 2010), cysteinyl leukotriene receptor 1 (CysLTR1) (Hui and Funk, 2002), and 11β-prostaglandin F2α (11β-PGF2α) (Ricciotti and FitzGerald, 2011)) in a population where different biomarker levels can be expected. The comparison with established urinary tests can help validate the breath test, which benefits from not requiring sample preparation and providing diagnostic results almost in real time.

Our primary aim was to assess the correlation of breath features with the urinary biomarkers of oxidative stress and airway inflammation in tobacco smoke-exposed and unexposed children, for whom we expect different concentrations of these biomarkers. Our secondary aim was to test the prediction of urinary biomarker concentrations by exhaled breath captured via real-time SESI-HRMS, which allows for the contemporaneous detection of a broad spectrum of exhaled features.

## Materials and methods

### Study population and design

In total, 48 children from smoking households and 112 children from non-smoking households were enrolled in this observational study between April 2018 and August 2021 (online Supplementary Figure S1). Children older than 18 years and those with acute asthma exacerbation, acute inflammatory disease, renal failure or renal replacement, or acute or chronic liver disease were excluded. The sample size was determined while controlling for the false discovery rate (FDR). Assuming a 5% FDR, a 0.7 expected standardized mean difference (Δ/σ) in breath (García-Gómez et al., 2016; Martinez-Lozano Sinues et al., 2014), and 90% power, a sample size of 50 subjects per arm was required. In accordance with the fraction of smokers in the population, twice as many children not exposed to ETS were recruited to ensure a contemporaneous assessment of exposed and non-exposed children. The Ethics Committee of Nordwestern and Central Switzerland approved the study (ID 2017–02038 and EKBB-Nr. 360/11), and written informed consent was obtained during enrollment.

### Breath samples

For each child, a breath sample was acquired via real-time SESI-HRMS in the same room, adhering to a standardized protocol (Gisler et al., 2022). Each measurement included six prolonged exhalations, both in positive and negative ionization mode. Children were instructed to remove cosmetics, fast, and refrain from chewing gum or using toothpaste for at least 1 h prior to the measurement to minimize confounding from these factors. The analytical platform consisted of an ion source (SUPER SESI, FIT Spain) coupled to a high-resolution mass spectrometer (Q Exactive Plus, Thermo Fisher Scientific, Germany) and an exhalation interface (Exhalion, FIT Spain) for the parallel assessment of CO_2_, flow rate, exhaled volume, and pressure drop. Commercially available bacterial filters (MicroGard, Vyaire Medical, United States) were used as mouthpieces. Mass spectra were acquired in the full scan mode over a range of m/z 100–400 with a resolution of 140,000 for positive and negative ionization mode. Two microscans, an automatic gain control (ACG) target of 1*10^6^, and a maximum injection time of 500 ms were applied. For electrospray formation, a 20-µm ID non-coated TaperTip silica capillary emitter (New Objective, Woburn, MA) and 0.1% formic acid in water were used. The SUPER SESI solvent reservoir pressure was set to 1.3 bar. The temperature of the ion chamber was set to 90°C, and the sampling line temperature was set to 130°C. To further minimize the adsorption of analytes on the system walls, the sampling line and the core of the ionization chamber were coated with silica. A stream of clean nitrogen (filtered through a built-in active charcoal filter) was used to flush the ion source between breath measurements. The exhaust mass flow controller was set at 0.7 L/min, and the nitrogen mass flow through the source was 0.35 L/min to ensure a constant fraction of breath entering the ionizer (0.3 L/min). Internal and external calibration of the mass spectrometer were performed regularly.

### Urinary samples

During the same visit when the breath sample was taken, a urine sample was collected. Urine samples were collected in 2-mL sterile microtubes (Sarstedt, Nümbrecht, Germany) and stored at −80°C until further analysis. Enzyme-linked immunosorbent assays (ELISAs) were used to assess the following: a) levels of cotinine (BioVision, United States) to classify ETS exposure (urine cotinine≥1800 pg/mg creatinine), b) levels of 8-iso-PGF2α (Cell Biolabs, United States), c) levels of CysLTR1 (Reddot Biotech, Canada), and d) levels of 11β-PGF2α (Cayman Chemicals, United States). The following limits of detection (LOD) were applied to the ELISAs (mean measured blanks + 3 standard deviations): cotinine, 2.864 ng/mL; 8-iso-PGF2α, 158.796 pg/mL; CysLTR1, 0.264 ng/mL; and 11β-PGF2α, 0.134 pg/mL. All ELISA kits were used according to the manufacturer’s protocols. For all urinary markers, a blank was subtracted from the measured concentration. Concentrations were set to 0 when blank concentration > measured concentration. All zeros were then replaced using regression on order statistics (ROS) using the NADA package in R. Consequently, creatinine levels assessed using ELISA (Invitrogen, Thermo Fisher, United States) were used to adjust the concentrations of all urinary biomarkers, which were then reported in units of pg/mg.

### Data analysis

Data preprocessing and statistical analyses were performed using MATLAB (version 2022a, MathWorks Inc., United States).

#### Preprocessing breath data

Untargeted analysis was performed for mass spectral breath data to evaluate the entire exhaled metabolic profile. RAW files were converted to MATLAB structure using Thermo’s RawFileReader. Peak alignment and mass calibration were ensured by i) performing internal and external calibration before data acquisition (lock masses and room air) and ii) further calibrating the mass spectra during post-acquisition using reference peaks. The feature list was generated from the centroid peak list via binning using ksdensity. Exhalation time windows were defined by CO_2_ concentrations above 2.5% (as measured by Exhalion). Subsequently, average mass spectra were computed for the corresponding exhalation scans using Thermo’s RawFileReader. Only peak centroids with signal intensity above 10 a.u. in Thermo’s signal intensity scale were selected to define the feature list. Subsequently, features occurring in >80% of the samples from ETS-exposed children were considered for further analysis. For those features, molecular formulas were assigned based on the accurate mass, considering the elements C, H, N, O, and S (Kind and Fiehn, 2007), and the following adducts: [H], [-H_2_O+ H], and [-H_2_O- H]. Zeros in the signal intensity of selected features were replaced using regression on order statistics using the NADA package in R.

#### Statistical tests

Correlations between urinary biomarkers themselves and between urinary biomarkers and breath features were calculated using Spearman’s correlation (ρ). Wilcoxon signed-rank tests were used to compare the levels of urinary biomarkers and breath profiles between ETS-exposed and non-exposed children. The false discovery rate (FDR) for multiple tests was controlled using the Benjamini–Hochberg (BH) method.

#### Prediction of urinary biomarkers

The breath-based prediction was evaluated for urinary biomarkers that revealed significant correlations (BH-adjusted *p*-values from Spearman’s correlation ≤0.01) with multiple breath features. For the prediction of urinary biomarker concentrations, we divided the study population into a training set (n = 90) and a test set (n = 38). To identify the best predictors, automatic variable selection was performed in the training set using the MATLAB algorithms ReliefF and TreeBagger. For the final prediction models, we screened 16 different regression models in the training set using the top 30 predictors identified in≥9000 out of 10,000 iterations. Finally, mean (± SD) Lin’s concordance correlation coefficient (CCC) (Lin, 1989) was calculated in the test set for the best-performing regression models to quantify the agreement between the actual and the breath-predicted urinary biomarker concentrations.

## Results

**FIGURE 1:**
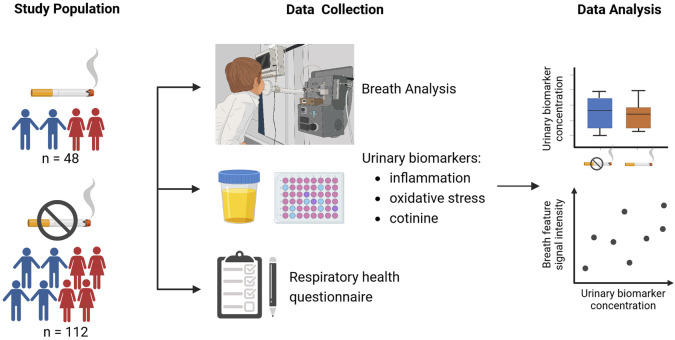
Overview of the study design (created with BioRender.com).

In total, 128 out of 160 children were included in the final analysis. From the final study sample of 128 children (online Supplementary Figure S1), 25 were exposed to ETS according to their cotinine level (urine cotinine≥1800 pg/mg creatinine). Details on the study design, study population, and urinary biomarker concentrations are outlined in Figure 1 and Table 1.

**TABLE 1:** Study population and distribution of urinary biomarkers.

	Total	ETS non-exposed^a^	ETS exposed^a^
Sample size	128	103	25
Self-reported ETS exposure, n (%)	41 (32)	18 (17)	23 (92)
Female individuals, n (%)	63 (49)	47 (46)	16 (64)
Age in years, mean (SD)	8.7 (3.5)	8.6 (3.5)	9.5 (3.6)
Cough this week, n (%)	28 (22)	18 (17)	10 (40)
Cough last 12 months, n (%)	68 (54)	60 (58)	8 (32)
Wheeze this week, n (%)	7 (6)	3 (3)	4 (16)
Wheeze last 12 months, n (%)	24 (20)	21 (20)	3 (12)
Inhalative steroids^b^ n (%)	11 (9)	7 (7)	4 (16)
Cotinine pg/mg creatinine, median (IQR)	197 (1069)	128 (290)	11,286 (17,667)
8-iso-PGF2α pg/mg creatinine, median (IQR)	1439 (1443)	1475 (1387)	1192 (1495)
11β-PGF2α pg/mg creatinine, median (IQR)	1056 (1009)	1028 (985)	1386 (976)
CysLTR1 pg/mg creatinine, median (IQR)	187 (564)	180 (539)	255 (671)

^a^ ETS non-exposed: urine cotinine <1800 pg/mg creatinine; ETS exposed: urine cotinine≥1800 pg/mg creatinine.

^b^ Number (%) of children who used inhalative steroids in the past 12 months.

**FIGURE 2:**
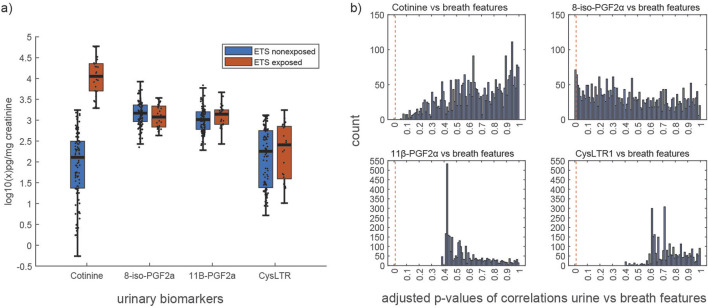
**(a)** Distribution of urinary biomarker concentrations in ETS exposed vs. non-exposed children. **(b)** Distribution of Benjamini–Hochberg (BH)-adjusted p-values from Spearman’s correlation of 3,126 breath mass spectral features and 4 urinary biomarkers (namely, cotinine, 8-iso-PGF2α, 11β-PGF2α, and CysLTR1); the red vertical line indicates the applied threshold for significance (adjusted p-value ≤0.01).

We identified 71 breath features that correlated significantly (BH-adjusted *p*-values from Spearman’s correlation ≤0.01) with the urinary oxidative stress marker 8-iso-PGF2α (online Supplementary Table S2). Of those breath features, 28 correlated positively and 43 correlated negatively with urinary 8-iso-PGF2α. We found no correlations between breath features and any of the other urinary biomarkers—cotinine, CysLTR1, 11β-PGF2α (Figure 2B; BH-adjusted *p*-values from Spearman’s correlation >0.01).

Because several breath features correlated significantly (BH-adjusted *p*-values from Spearman’s correlation ≤ 0.01) with urinary 8-iso-PGF2α, we tested the prediction of this biomarker by exhaled breath. The agreement (mean (± SD) Lin’s CCC) between breath-predicted and actual concentrations of urinary 8-iso-PGF2α was 0.37 (0.05).

Concentrations of urinary 8-iso-PGF2α, CysLTR1, and 11β-PGF2α did not correlate with those of urinary cotinine (Spearman’s correlation *p*-values >0.05; online Supplementary Table S1) and consequently did not differ between ETS-exposed and non-exposed children (Figure 2A; *p*-values from Wilcoxon signed-rank test: 8-iso-PGF2α = 0.287, 11β-PGF2α = 0.160, and CysLTR1 = 0.333). We also found that the breath profiles of ETS-exposed and non-exposed individuals did not differ (BH-adjusted p-values from the Wilcoxon signed-rank test >0.05).

## Discussion

In this study, we analyzed full breath profiles of ETS-exposed and non-exposed children alongside established urinary biomarkers of oxidative stress and airway inflammation to evaluate the correlation between the two modalities. We found multiple breath features correlating with the urinary oxidative stress marker 8-iso-PGF2α and showed that this biomarker could be predicted reasonably well by exhaled breath. To the best of our knowledge, this is the first study to evaluate correlations between the concentrations of urinary oxidative stress and airway inflammation markers and exhaled breath profiles acquired via real-time SESI-HRMS.

We found no correlations between the urinary biomarkers (8-iso-PGF2α, 11β-PGF2α, and CysLTR1) and the level of ETS exposure, as measured by the creatinine-adjusted cotinine level. We assume that, in general, the ETS exposure was too low (median cotinine in ETS-exposed group: 11,286 pg/mg creatinine) to cause an effect that is measurable in the urine. Nevertheless, and independent of ETS exposure, we observed low but varying concentrations of the oxidative stress marker 8-iso-PGF2α in our study population. Interestingly, even these very low concentrations of 8-iso-PGF2α correlated significantly with the signal intensity of 71 breath features. We assume that this correlation can also be found for clinically relevant concentrations of this oxidative stress marker.

In addition, we observed that urinary 8-iso-PGF2α concentrations can be predicted reasonably well (mean Lin’s CCC: 0.37) by several metabolites in breath. These findings support the previously identified potential of SESI-HRMS to gauge oxidative stress non-invasively and in real-time (García-Gómez et al., 2015). To further evaluate these results, a study in a population with a broader range of oxidative stress levels, including children with typically elevated concentrations (e.g., pulmonary diseases), is needed. We did not detect correlations between breath and the airway inflammation markers 11β-PGF2α and CysLTR1. Possibly, the signal intensity of the correlating metabolites in the breath was too low, or the corresponding metabolites were not volatile and therefore could not be detected in exhaled breath.

The limitations of this study include i) a thorough compound identification for correlating breath features that remains to be accomplished. ii) The prediction of urinary biomarkers by exhaled breath has not been externally validated in an independent cohort of children.

In conclusion, our results suggest that the real-time breath analysis via SESI-HRMS has promising potential as an adjunct to existing diagnostic methods for monitoring oxidative stress, even at relatively low levels. Our findings may open new opportunities for the non-invasive detection of respiratory disease, which is particularly relevant to the pediatric population.

## Data Availability

The raw data supporting the conclusions of this article will be made available by the authors, without undue reservation.
