# Supplementary material for: Urinary marker of oxidative stress in children correlates with molecules in exhaled breath
Source: Front Mol Biosci. 2025 Mar 26;12:1511119. doi: 10.3389/fmolb.2025.1511119 (PMC11978638; doi:10.3389/fmolb.2025.1511119)
Supplement: Supplementary file 1 [file DataSheet1.docx]

**Online Supplement**

**Urinary marker of oxidative stress in children correlates with molecules in exhaled breath**


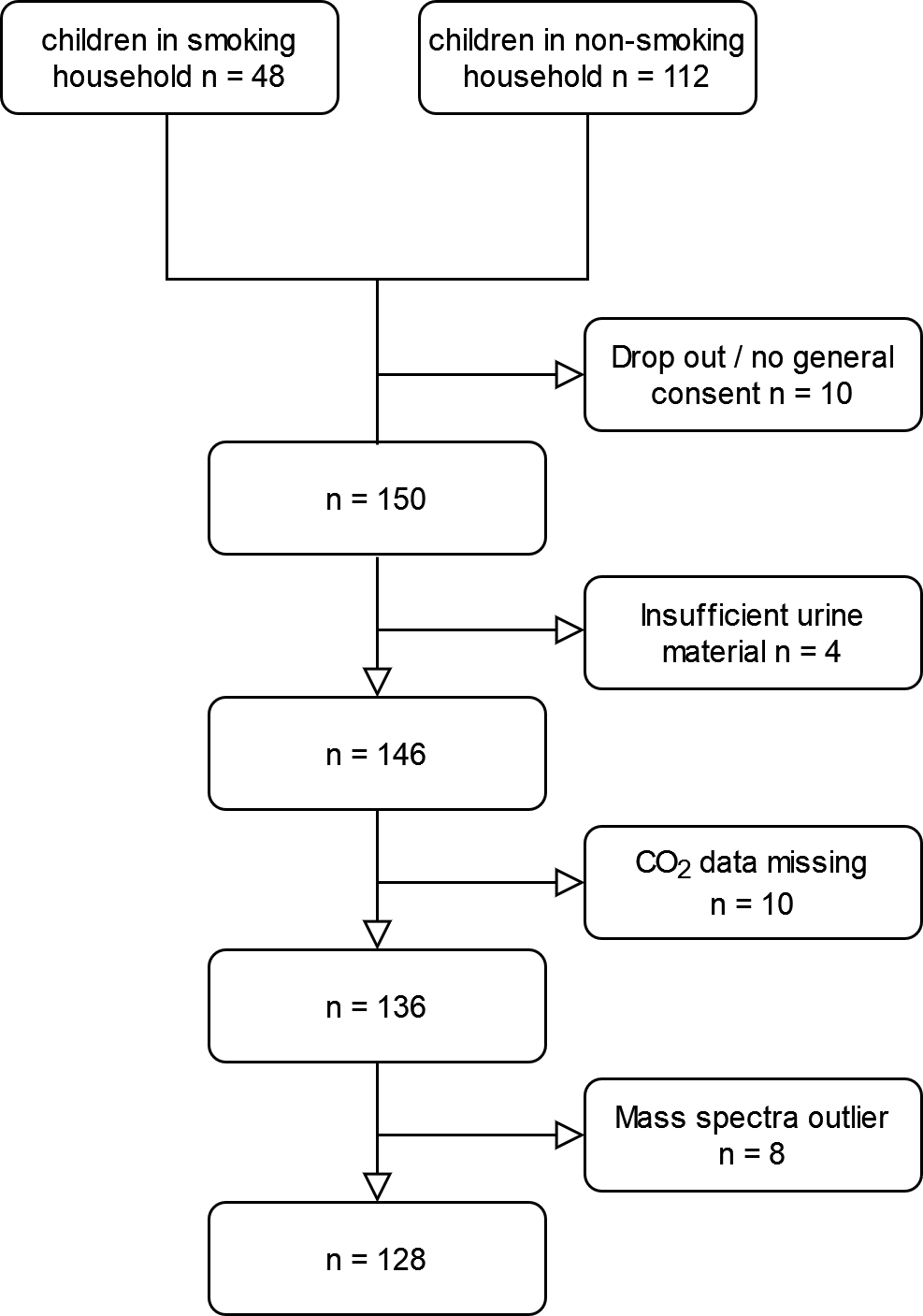


**Figure S1** Flowchart study population. Abbreviations: CO_2_ - Carbon dioxide.

| **Table S1** Spearman correlation coefficients of urinary biomarker concentrations | | | | |
| --- | --- | --- | --- | --- |
|  | Cotinine | 8-iso-PGF2α | 11β-PGF2α | CysLTR1 |
| Cotinine | 1.0 | *0.09* | *0.08* | *0.34* |
| 8-iso-PGF2α | -0.2 | 1.0 | *0.89* | *0.26* |
| 11β-PGF2α | 0.2 | 0.0 | 1.0 | *0.85* |
| CysLTR1 | 0.1 | -0.1 | 0.0 | 1.0 |
| Italic numbers shaded in grey represent p-values from the spearman correlation | | | | |

| **Table S2** Breath features significantly (adjusted p-value ≤ 0.01) correlating with urinary 8-iso-PGF2α | | | | |
| --- | --- | --- | --- | --- |
| **Exact Adduct Mass** | **Molecular Formula^a^** | **Mode** | **BH adjusted p-value** | **Spearman’s rho (ρ)** |
| 101.0608 | C_5_H_10_O_2_ | negative | 2.99E-03 | -0.36 |
| 102.0642 | na | negative | 2.99E-03 | -0.36 |
| 103.0651 | na | negative | 7.33E-04 | -0.40 |
| 104.0454 | na | positive | 8.38E-04 | -0.39 |
| 111.0917 | C_6_H_12_ON_2_ | positive | 9.85E-03 | 0.32 |
| 127.0230 | na | negative | 5.17E-03 | -0.34 |
| 133.0679 | C_6_H_14_O_2_S | positive | 3.32E-03 | 0.35 |
| 134.0675 | na | positive | 4.88E-03 | 0.34 |
| 141.1135 | C_6_H_14_ON_4_ | positive | 3.59E-03 | -0.35 |
| 147.0200 | C_7_H_4_O_2_N_2_ | negative | 3.60E-03 | -0.35 |
| 149.0118 | na | negative | 4.16E-04 | -0.41 |
| 149.0346 | C_7_H_6_O_3_N_2_ | positive | 6.70E-03 | -0.34 |
| 149.0808 | C_6_H_14_O_5_ | positive | 6.70E-03 | 0.33 |
| 150.0186 | C_7_H_5_O_4_N | positive | 1.80E-03 | -0.37 |
| 151.0965 | C_6_H_16_O_5_ | positive | 1.06E-03 | 0.39 |
| 152.0998 | na | positive | 7.52E-04 | 0.40 |
| 174.0913 | C_11_H_13_O_2_N | positive | 1.97E-03 | -0.37 |
| 176.1645 | C_9_H_23_O_3_N | positive | 4.11E-04 | -0.41 |
| 178.0499 | C_9_H_9_O_4_N | positive | 9.38E-03 | -0.32 |
| 179.0637 | C_9_H_12_ON_2_S | positive | 1.06E-03 | -0.39 |
| 179.0914 | C_7_H_16_O_6_ | positive | 2.73E-04 | 0.42 |
| 180.0671 | C_10_H_7_N_5_ | positive | 1.06E-03 | -0.39 |
| 183.2107 | C_13_H_28_O | positive | 2.19E-03 | 0.36 |
| 186.1852 | C_11_H_25_O_2_N | positive | 6.67E-03 | -0.34 |
| 187.1886 | C_12_H_28_S | positive | 1.37E-03 | -0.38 |
| 190.0510 | C_10_H_9_O_3_N | negative | 7.42E-03 | -0.33 |
| 191.0855 | C_15_H_12_O | positive | 8.24E-04 | 0.40 |
| 192.0889 | C_8_H_11_O_2_N_5_ | positive | 1.09E-03 | 0.38 |
| 195.1227 | C_8_H_20_O_6_ | positive | 2.00E-03 | 0.37 |
| 196.1106 | na | negative | 4.30E-03 | -0.35 |
| 196.1261 | C_10_H_19_N_3_S | positive | 1.25E-03 | 0.38 |
| 204.2039 | na | positive | 3.85E-03 | -0.35 |
| 209.1384 | C_9_H_22_O_6_ | positive | 2.41E-04 | 0.43 |
| 214.2529 | C_14_H_33_ON | positive | 2.32E-03 | 0.36 |
| 217.1809 | C_12_H_26_O_3_ | negative | 1.26E-03 | -0.38 |
| 219.1227 | C_10_H_20_O_6_ | positive | 7.42E-03 | 0.33 |
| 219.1955 | C_12_H_28_O_4_ | positive | 9.98E-03 | 0.32 |
| 220.1458 | na | positive | 5.10E-03 | -0.34 |
| 221.1384 | C_10_H_22_O_6_ | positive | 1.11E-03 | 0.38 |
| 223.1176 | C_9_H_20_O_7_ | positive | 3.10E-04 | 0.42 |
| 223.1540 | C_10_H_24_O_6_ | positive | 2.03E-04 | 0.44 |
| 231.1591 | C_12_H_24_O_5_ | positive | 1.06E-03 | 0.39 |
| 231.2319 | C_14_H_32_O_3_ | positive | 3.56E-04 | 0.42 |
| 232.2352 | C_8_H_27_N_9_ | positive | 1.11E-03 | 0.38 |
| 233.1384 | C_11_H_22_O_6_ | positive | 3.75E-03 | 0.35 |
| 234.1852 | C_15_H_25_O_2_N | positive | 7.10E-03 | -0.33 |
| 239.1489 | C_10_H_24_O_7_ | positive | 8.12E-04 | 0.40 |
| 245.2111 | C_14_H_30_O_4_ | positive | 1.06E-03 | 0.39 |
| 247.2067 | C_17_H_28_O | negative | 3.10E-04 | -0.42 |
| 249.1697 | C_12_H_26_O_6_ | positive | 2.60E-04 | 0.43 |
| 251.1853 | C_12_H_28_O_6_ | positive | 3.60E-03 | 0.35 |
| 258.1781 | C_8_H_21_O_2_N_9_ | positive | 8.68E-03 | -0.33 |
| 259.2268 | C_15_H_32_O_4_ | positive | 9.98E-03 | 0.32 |
| 273.2424 | C_16_H_34_O_4_ | positive | 8.02E-05 | 0.46 |
| 275.2581 | C_16_H_36_O_4_ | positive | 3.07E-05 | 0.49 |
| 279.2682 | C_19_H_36_O_2_ | positive | 9.38E-03 | 0.32 |
| 281.2839 | C_19_H_38_O_2_ | positive | 7.19E-03 | 0.33 |
| 281.3203 | C_20_H_42_O | positive | 6.78E-03 | 0.33 |
| 283.0304 | C_9_H_16_O_7_S_2_ | positive | 2.52E-03 | 0.36 |
| 287.2217 | C_16_H_32_O_5_ | positive | 1.06E-03 | 0.39 |
| 287.2581 | C_17_H_36_O_4_ | positive | 1.11E-03 | 0.38 |
| 293.2839 | C_20_H_38_O_2_ | positive | 5.42E-03 | 0.34 |
| 302.2418 | C_11_H_29_ON_9_ | negative | 8.68E-03 | -0.33 |
| 303.2530 | C_17_H_36_O_5_ | positive | 2.60E-04 | 0.43 |
| 303.2894 | C_18_H_40_O_4_ | positive | 8.23E-05 | 0.46 |
| 315.0564 | C_22_H_8_ON_2_ | negative | 1.93E-04 | 0.44 |
| 317.2639 | C_10_H_30_N_12_ | negative | 1.25E-03 | -0.38 |
| 317.2686 | C_18_H_38_O_5_ | positive | 5.15E-03 | 0.34 |
| 323.3308 | C_22_H_44_O_2_ | positive | 9.77E-03 | 0.32 |
| 330.2731 | C_13_H_33_ON_9_ | negative | 8.59E-03 | -0.33 |
| 331.2843 | C_19_H_40_O_5_ | positive | 4.30E-03 | 0.35 |
| a Molecular formula assigned through MATLAB using seven golden rules^1^ | | | | |

**References**

1. Kind T, Fiehn O. Seven Golden Rules for heuristic filtering of molecular formulas obtained by accurate mass spectrometry. *BMC Bioinformatics.* 2007;8:105.
